# Patents *Versus* No Patents

**Published:** 1898-10

**Authors:** G. Alden Mills

**Affiliations:** New York


### PATENTS *VERSUS* NO PATENTS.

BY DR. G. ALDEN MILLS, NEW YORK.

At the meeting of the New York Institute of Stomatology, Dr. St. John Roosa stated that medical men say that dentists have no code of ethics. Of course, such a charge will irritate many of a certain kind. We all know that a man in temper is weak, and in that proportion his influence is lost.

When self-interest most predominates the written code may seem to hold men to ethical conduct, but there are a multitude of tricks that can be resorted to that would manifest anything but an ethical character.

Another statement Dr. Roosa made at this meeting, which has great weight, is that the medical profession has kept absolutely free from the taking out of patients, and that it has not been debarred from all the benefits that have been devised for its advantage and for the amelioration of the ills of humanity. Have not the members of this profession gained as much in value by a professional *esprit du corps* as our profession by the opposite course? Have we not placed commerce against professional spirit? and, again, has the medical profession not gained as much? We cannot gainsay the fact that the members of this profession are far better established in the matter of fees than we are. The public regard them professionally in a far higher sense than they do us. They do not question them regarding the remedies they use, nor dictate as to what they shall prescribe. They do not solicit them for outside operations. The aim of the public is to get the benefit of their skill. The medical profession is not hounded by courts at law for the collection of royalties. We have seemingly been the creatures of misfortunes which date back to the Vulcanite Company. It is not necessary to repeat the history, it is too bitterly fastened upon the memory of those who suffered. The millions that were so mercilessly wrested from dentists stirred other agents of his Satanic Majesty to try and repeat the experiment, but they lost their crown and had all their bridges destroyed. No words of praise are too strong to express the gratitude that exists in the hearts of many regarding the labor and the results obtained by Dr. Crouse in our behalf. Such high-minded and unflinching purpose has its record in the annals of dentistry.

It is recorded that the "love of money is the root of all evil." This was quoted to a friend, and he rejected it and said, "It was not true," but, "God is true though all men be liars."

This brings us to the thought, may it not be that the inordinate love for getting has burdened our profession with barnacles of selfishness. It is not to be expected that a way will be devised that will liberate us as a whole, but it is believed it will result in a division of interest. There is an aim worthy of emulation directing to a higher professional standing in practice, which can only come through a larger mental grasp of what is true. Money is power, and this is showing itself in every direction in human affairs.

What has proved true in the medical profession without patents is that the public has a more exalted opinion of their worth, and so far as the dental profession gravitates towards the mercantile atmosphere will we be measured by the spirit of such an environment, and more and more will the professional spirit be lost. The mass of practitioners will gravitate to a business basis, and such a thing cannot be sustained on true professional grounds. There need not, however, be any warfare, simply drop those who prefer this course into the line of their own selection. There will continue to be a support for those who aim for high attainments, and to those will come the reward of appreciation. Skill for bodily comfort by remedial agencies in medicine and surgery cannot be placed in the scale of finance for adjustment. True professional practitioners will not be driven from the path of rectitude nor disturbed by the competition of business.

It is not true that the day has gone by for liberal fees, never was there a more favorable period for the reward of professional skill. We are coming to see this as never before. Through experience it has been a cause of wonder whether there could be a just recognition for faithful service, but the clouds are being dispelled by practical demonstrations. It is not possible for the writer to march with the croakers. The days, professionally, are no longer clouded by doubts. Having aimed for the highest the "patent" is not desired. "What have we that we have not received. Who maketh us to differ?"

